# Target Antigen Attributes and Their Contributions to Clinically Approved Antibody-Drug Conjugates (ADCs) in Haematopoietic and Solid Cancers

**DOI:** 10.3390/cancers15061845

**Published:** 2023-03-19

**Authors:** Benjamina Esapa, Jiexuan Jiang, Anthony Cheung, Alicia Chenoweth, David E. Thurston, Sophia N. Karagiannis

**Affiliations:** 1 St. John’s Institute of Dermatology, School of Basic & Medical Biosciences, King’s College London, Guy’s Hospital, London SE1 9RT, UK; 2 Breast Cancer Now Research Unit, School of Cancer & Pharmaceutical Sciences, King’s College London, Guy’s Cancer Centre, London SE1 9RT, UK; 3 Institute of Pharmaceutical Science, School of Cancer and Pharmaceutical Sciences, King’s College London, London SE1 9NH, UK

**Keywords:** monoclonal antibodies, mAb, antibody-drug conjugates (ADCs), Fab regions, antigen, target, effector functions, IgG, checkpoint inhibitors, cancer immunotherapy

## Abstract

**Simple Summary:**

Antibody-Drug Conjugates (ADCs) provide effective anti-cancer treatments. ADC development requires the identification of appropriate tumour-associated antigens that can be targeted by the ADC to effectively kill cancer cells while minimising damage to healthy cells, thus limiting systemic toxicities. In this review, we examine the attributes of the antigens targeted by the anticancer ADCs that are clinically approved, and consider how these features may contribute to the safety and effectiveness of ADC therapeutics.

**Abstract:**

Antibody drug conjugates (ADCs) are powerful anti-cancer therapies comprising an antibody joined to a cytotoxic payload through a chemical linker. ADCs exploit the specificity of antibodies for their target antigens, combined with the potency of cytotoxic drugs, to selectively kill target antigen-expressing tumour cells. The recent rapid advancement of the ADC field has so far yielded twelve and eight ADCs approved by the US and EU regulatory bodies, respectively. These serve as effective targeted treatments for several haematological and solid tumour types. In the development of an ADC, the judicious choice of an antibody target antigen with high expression on malignant cells but restricted expression on normal tissues and immune cells is considered crucial to achieve selectivity and potency while minimising on-target off-tumour toxicities. Aside from this paradigm, the selection of an antigen for an ADC requires consideration of several factors relating to the expression pattern and biological features of the target antigen. In this review, we discuss the attributes of antigens selected as targets for antibodies used in clinically approved ADCs for the treatment of haematological and solid malignancies. We discuss target expression, functions, and cellular kinetics, and we consider how these factors might contribute to ADC efficacy.

## 1. Key Features of ADCs

### 1.1. Introduction to Antibody Therapeutics in Oncology

The development of antibody-based therapeutics has transformed the field of clinical oncology, in which the traditional mainstays such as surgery, chemotherapy, and radiotherapy are limited by factors such as the poor accessibility of tumours and the systemic toxicity of non-specific treatments [1,2,3]. The advent of monoclonal antibody (mAb) therapies allowed for the potential circumvention of these limitations, providing tumour-targeted treatments that might reduce toxic side effects compared to radiotherapy and chemotherapy [1,4,5]. The first mAb to be approved by the U.S. Food and Drug Administration (FDA) was the human/mouse chimeric IgG1 antibody rituximab, specific for the cell surface B-cell marker CD20. Rituximab was approved in 1997 for the treatment of non-Hodgkin lymphoma (NHL) [6]. This was followed by the 1998 FDA approval of trastuzumab, a humanised monoclonal antibody targeting the tumour antigen HER2, for the treatment of HER2-positive breast cancers [7]. These approvals provided the foundation for the development of many more antibodies and antibody scaffolds for several malignant diseases. At present, more than forty monoclonal antibody therapies have been approved by the FDA for the treatment of haematological malignancies and solid tumours [1].

A monoclonal antibody designed for cancer therapy can engender a range of antitumour mechanisms. Via its high specificity and affinity for a specific epitope of a target antigen, an antibody can exert direct Fab-mediated effects on a target-expressing cell by interfering with the antigen’s signalling functions or by blocking the target’s interaction with its ligands [1]. These attributes can impair important processes that support cancer cell survival, growth, and metastasis. The Fc regions can also be harnessed or manipulated to influence an antibody’s engagement with immune effector cells, which express cognate Fc receptors to engage and activate the immune system [8,9,10]. Monoclonal antibodies in clinical use can target several different entities, including cancer-associated antigens, cancer-associated vasculature, or checkpoint molecules on immune cells [1].

### 1.2. Antibody-Drug Conjugates (ADCs)

**Figure 1:**
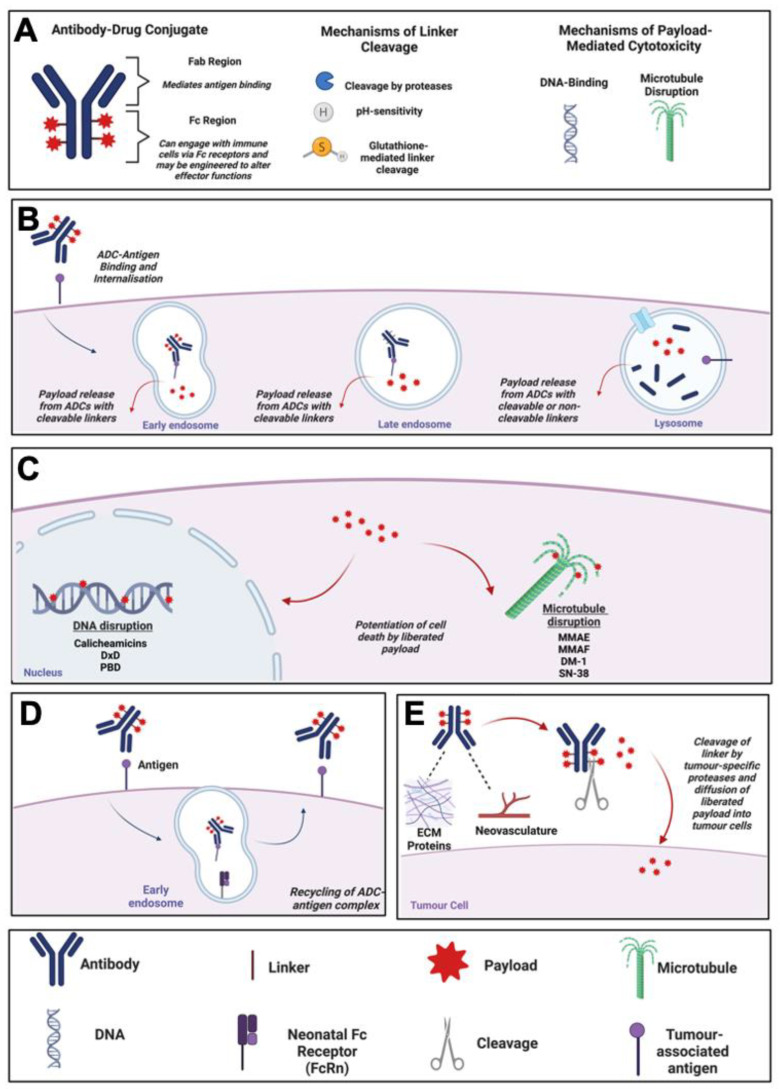
ADC Mechanism of Action. (**A**) Schematic diagram of an ADC, and descriptions of mechanisms of linker cleavage and payload toxicity. (**B**) Mechanism of action of an internalising ADC: Internalisation of ADC, trafficking to early and late endosomes and lysosomes followed by release of payload. (**C**) Mechanisms of cell death potentiated by ADC payloads, and relevant examples. (**D**) Schematic diagram of ADC recycling mediated by the neonatal Fc receptor (FcRn). (**E**) Mechanism of action of a non-internalising ADC. ADC binding to tumour-proximal extracellular matrix proteins or neovasculature before linker cleavage by proteases and release of cytotoxic payload. Created using Biorender.com. Abbreviations: ADC, Antibody-Drug Conjugate; DM-1, Mertansine DM-1; Dxd, Deruxtecan; ECM, Extracellular Matrix; FcRn, Neonatal Fc Receptor; MMAE/F, Monomethyl Auristatin E/F; PBD, Pyrrrolobenzodiazepine.

Antibody-based therapies have been developed in the form of antibody-drug conjugates (ADCs) and immunotoxins, taking advantage of the high affinity and selectivity of an antibody for a specific epitope on the target antigen to deliver a payload to the tumour site [2,4,11,12,13,14,15]. Immunotoxins are protein-based conjugates consisting of a target-binding whole antibody or antibody fragment for mediating target localisation, conjugated to a protein toxin usually derived from plant or bacterial species via gene fusion [16]. ADCs are therapeutics consisting of an antibody and a cytotoxic drug payload with inherent antitumour activity, joined through a chemical linker (Figure 1A) [2,4].

Building on the success of mAb therapeutics, ADCs combine the specificity of an antibody with the cytotoxicity of a drug-like payload to selectively target and kill malignant cells, theoretically sparing healthy cells and allowing for the administration of powerful cytotoxic agents that would ordinarily be too toxic to be delivered alone systemically [2]. The field of ADCs has grown rapidly since the first ADC, gemtuzumab ozogamicin (Mylotarg^®,^), a humanised IgG4 antibody targeting CD33 and conjugated to the DNA-cleaving agent calicheamicin, was approved by the FDA in 2000 for the treatment of CD33-expressing acute myeloid leukaemia (AML) [17]. There are presently twelve FDA- approved drugs (eight of which are also approved by the European Medicines Agency, EMA) for the treatment of solid tumours and haematological malignancies.

In addition, one HER2-targeted ADC, disatamab vedotin (Aidixi^®^), was approved in China in 2021 for the treatment of gastrointestinal (GI) and urothelial carcinomas, and two immunotoxins, tagraxofusp-erzs (Elzonris^®^) and moxetumomab pasudotox (Lumoxiti^®^) were approved by the FDA in 2018. The ADCs approved by the FDA and EMA are summarised in Table 1, and a selection of ADCs that are in late-stage clinical development are shown in Table 2.

### 1.3. Mechanism of Action of ADCs

The canonical mechanism of action of ADCs has been well-characterised and involves binding of the antibody to its respective antigen on target cells before internalisation of the ADC by receptor-mediated endocytosis. Once internalised, antigen-ADC complexes in endosomes fuse with lysosomes (Figure 1B). Liberation of ADC payloads requires cleavage of the linkers at tumour sites, with systemic cleavage of ADC linkers minimised, thus theoretically avoiding serious systemic toxicities [18]. Linker cleavage may be triggered by three major mechanisms. First, ADCs that are internalised by tumour cells may employ linkers susceptible to cleavage by intracellular-acting proteases that may be overexpressed in the tumour cells [18]. Second, acid-labile linkers may be employed, which are cleaved by acidic conditions in the lysosomes post-internalisation. Third, linkers may be cleaved intracellularly by thiol-disulphide bond exchange potentiated by thiols such as glutathione, which are expressed more highly in cancer cells compared with non-malignant cells [18,19].

On the other hand, ADCs with non-cleavable linkers depend entirely on internalisation followed by complete lysosomal proteolytic degradation of the entire conjugate to release the toxic payload [20]. Non-cleavable linkers offer the advantage of increased plasma stability of the ADC complex, thus reducing the likelihood of premature payload release. This could potentially provide a larger therapeutic window and greater tolerance compared to ADCs with cleavable linkers [21].

Depending on the payload targets, which most often are tubulin or DNA, the liberated cytotoxic payload then leads to microtubule disruption in the cytosol or DNA damage in the nucleus, ultimately resulting in tumour cell death via apoptosis [3] (Figure 1C). An alternative trafficking pathway, the up-regulation of which is considered to represent a potential resistance mechanism against ADCs, involves the recycling of endocytosed antigen-ADC complexes back to the plasma membrane, which may be mediated by binding of the ADC to the neonatal Fc (FcRn) receptor in the early endosome (Figure 1D) [2,22].

A further mechanism of ADC-mediated cytotoxicity is the so-called “bystander effect”, in which cells surrounding the target cancer cell may be killed independently of target antigen expression due to the payload detaching from the ADC in the extracellular space or following free payload release out of the cytosol following ADC internalisation and lysosomal processing. These processes result in the unconjugated payload diffusing into surrounding cells and potentiating their death in the tumour microenvironment (TME) [11,23]. Additionally, non-internalising ADCs directed towards non-cellular components of the tumour microenvironment, such as the neovasculature or extracellular matrix proteins up-regulated during cancer, may be designed using linkers that are specifically cleaved by tumour-specific proteases (Figure 1E). These ADCs are designed to potentiate the extracellular release of membrane-permeable cytotoxic payloads specifically at tumour sites [24]. Examples of non-internalising ADCs evaluated in pre-clinical studies are F16-MMAE and F16-PNU159682, which comprise an IgG1 antibody (F16) targeting the splice variant of tenascin-C, a glycoprotein of the extracellular matrix that is expressed in the stroma of several lymphomas, conjugated via cleavable linkers to MMAE or the anthracycline derivative PNU159682, respectively [25,26]. Evaluation of these ADCs in a nude mouse model of squamous cell carcinoma found that both restricted the growth of xenografts, with F16-PNU159682 showing higher potency at a lower dose than F16-MMAE, albeit with significant toxicities [26].

### 1.4. Classes of ADC Payloads

The design of ADCs requires the selection of the most appropriate payload. Seven out of the twelve approved ADCs are designed with payloads that inhibit microtubule polymerisation, mostly based on auristatin or maytansinoid molecules. Other payload classes include calicheamicins (DNA cleaving), IGNs (DNA guanine mono-alkylating), duocarmazine (DNA adenine mono-alkylating), pyrrolobenzodiazepine dimers (PBDs) (DNA guanine-guanine cross-linking), and camptothecin-type derivatives (topoisomerase inhibiting). The payload classes used in the currently approved FDA- and EMA-approved ADCs are briefly described below. Payloads with DNA guanine mono-alkylating (i.e., the IGNs) and DNA adenine mono-alkylating (i.e., duocarmazine) mechanisms of action are found in ADCs in late-stage clinical development (see Table 2), but no ADCs containing them are yet approved.

#### 1.4.1. Tubulin Inhibitors

A class of ADC payload that functions through tubulin inhibition comprises compounds of the maytansinoid or “DM” family, which have been extensively used for the development of ADCs. DMs are derived from the macrolide maytansine, which was isolated from the *Maytenus ovatus* shrub in the 1970s [27]. DMs work by binding to tubulin and blocking its polymerisation, thus leading to mitotic arrest [28]. The most widely used ADC containing a maytansinoid payload is trastuzumab emtansine, which comprises a humanised anti-HER2 IgG1 antibody conjugated to emtansine (DM-1).

The auristatins are the other main payload class that functions through tubulin inhibition. They are derivatives of a family of naturally occurring structures known as dolostatins, which are cytotoxins extracted from marine molluscs in the 1970s. Dolostatin 10 was found to bind to tubulin and, after limited success as stand-alone agent in anti-cancer clinical trials, the water-soluble derivatives monomethyl auristatin E (MMAE) and monomethyl auristatin F (MMAF) were developed. These agents exhibited potent cytotoxicity against cancer cells and the ability to induce immunogenic cell death [29].

One advantage of MMAE is its membrane permeability, which means it can exert bystander killing when conjugated to antibodies, as evident from numerous in vitro studies [30,31]. MMAF has a comparatively reduced membrane permeability due to the presence of a C-terminal phenylalanine residue, which confers a negative charge on the molecule [30,32]. Interestingly, structural studies of MMAE and MMAF suggest that the conformations of these molecules found most abundantly in solution may not be tubulin interactive. There are two conformational states, *cis* and *trans* isomers, with the *cis* isomer being the predominant species present in solution, distinct from the reported tubulin-binding trans structure. Therefore, there may be scope to increase the potency of the auristatins through chemical design to ensure that they are in the biologically active conformation [33]. Auristatin-mediated keratopathy was observed in the DREAMM-2 Phase II clinical trial which evaluated the MMAF-conjugated ADC belantamab mafodotin-blmf (Blenrep^®^) for the treatment of multiple myeloma [34]. Chemical modification of auristatins to increase potency and allow administration of lower doses may therefore reduce auristatin-mediated toxicities.

#### 1.4.2. Topoisomerase Inhibitors

Two approved ADCs utilise payloads that function through topoisomerase inhibition. One of these is sacituzumab govitecan (Trodelvy^®^), an anti-TROP-2 IgG1 antibody conjugated to SN-38, and the other is trastuzumab deruxtecan (Enhertu^®^), an anti-HER2 IgG1 antibody conjugated to deruxtecan (Dxd). SN-38 and Dxd are both related to the plant alkaloid camptothecin, and inhibit topoisomerase enzymes essential for the relaxation of supercoils during DNA replication, with Dxd being a higher-potency analogue of SN-38 [35,36,37]. Inhibition of topoisomerase function leads to DNA strand breaks during replication, followed by cell death through apoptosis [36]. In vitro studies indicate that Dxd is membrane-permeable, with ADCs containing it producing a significant bystander effect [38]. 

#### 1.4.3. DNA Cross-Linking Agents

DNA cross-linking payloads such as the pyrrolobenzodiazepine dimers (PBD dimers) bind in the minor groove of DNA, forming inter- and intra-strand cross-links between two guanine base pairs (as well as a lower level of mono-alkylated adducts). The adducts formed can prevent strand separation, with attempts at DNA repair leading to strand cleavage and cell death through apoptosis [39]. The suitability of PBD dimers as ADC payloads is exemplified by the FDA approval of loncastuximab tesirine (Zynlonta^®^), an anti-CD19 ADC used for the treatment of relapsed or refractory diffuse large B-cell lymphoma (DLBCL) [40]. As for some of the tubulin-inhibiting and topoisomerase-inhibiting payloads described above, ADCs containing a PBD dimer as a payload have been reported to produce a significant bystander effect [41].

#### 1.4.4. DNA-Cleaving Agents

Calicheamicin is an ADC payload that works by binding in the minor groove of DNA and causing double-strand breaks of the DNA helix through the backbone sugars. This leads to cell cycle arrest at the G2/M phase, followed by cell death through apoptosis [42]. This results in a very high level of cytotoxicity in tumour cell lines. A calicheamicin derivative was first used in gemtuzumab ozogamicin (Mylotarg^®^), an anti-CD33 IgG4 antibody approved by the FDA in 2000 for the treatment of ALL [17]. This ADC was subsequently withdrawn from clinical use in 2010 due to a lack of convincing efficacy data, but was subsequently fully re-approved in 2017. The same payload is used in inotuzumab ozogamicin (Besponsa^®^), an anti-CD22 IgG4 ADC approved by the FDA in 2017 for the same disease [43].

## 2. Features of Antigens for Antibody Target Selection in ADC Design

The use of highly potent cytotoxic agents in ADCs necessitates judicious target antigen selection aimed at maximising tumour selectivity and antitumour potency while minimising on-target off-tumour dose-limiting toxicities. Therefore, delivering a payload as specifically as possible to cancer cells is of paramount importance for ADC design while attempting to balance factors such as safety and efficacy. Tumour-specific targets can be identified for more than one specific cancer type and sometimes for a range of malignancies [44].

According to the currently accepted dogma, the ideal target antigen for an effective ADC should be expressed with sufficient density and homogeneity on the surface of tumour cells, and with minimal expression on normal cells, to limit on-target off-tumour toxicity and to optimise the therapeutic index [14,44,45]. The majority of preclinical and clinical targets for ADCs are tumour-associated rather than tumour-specific [44], meaning that they are overexpressed by cancer cells and expressed to some extent by normal cells [12,45]. Therefore, an important consideration is whether ADC targets are present and, if so to what extent, in vital or regenerative non-malignant tissues [23,46].

In addition to specific and abundant expression, an optimal target antigen also needs to be extracellular in order for the mAb to access the antigenic epitope [13,15,22,44]. Furthermore, it is accepted that ADC efficacy usually depends on efficient target-mediated internalisation to deliver the cytotoxic payload inside cancer cells, thus making internalisation following mAb binding a particularly important property for ADC target antigens. The internalisation rate and kinetics of endosomal-lysosomal trafficking upon tumour antigen binding to an ADC may therefore directly influence efficient payload release and cancer cell killing [47]. It is also critical to understand whether an antigenic target is predominantly directed to a recycling or lysosomal-targeted pathway [22]. The recycling of antigen-ADC complexes to the plasma membrane is thought to compromise efficient delivery of ADCs to the lysosomes and may impede the release of the payload to the cytosol and consequently impair the potency of the ADC [48,49]. A further factor with implications for the effectiveness of ADCs is the rate of removal of an antigen from the cell surface, often mediated by proteases produced by the tumour cells, in a process known as antigen shedding [50,51,52,53].

Important target antigens for both haematological and solid cancers are described in the following sections.

### 2.1. ADC Targets for Haematological Cancers

There are many FDA and EMA-approved ADCs that target haematological malignancies [54]. Due to the large number of circulating tumour cells, haematological cancers are considered to be generally more accessible and thus more easily treated compared to solid tumours in which it may be more challenging for therapeutic agents to penetrate to reach cancer cells at the centre [55]. Therefore, ADCs, which are normally introduced into the patient’s circulation, have immediate access to circulating malignant haematological cells. However, ADCs designed to target solid tumours need to extravasate, penetrate tissues, and navigate the tumour microenvironment (TME) in order to exert their antitumour effects.

For haematological cancers, immune lineage-specific biomarkers such as CD19, CD20, CD22, CD33, BCMA (B-cell maturation antigen), and CD79 are broadly and homogenously expressed on malignant haematological cells at high levels, and so have been extensively explored as candidate targets for ADC development [4]. However, the same antigens are often expressed on their equivalent non-malignant cell counterparts in the circulation and lymphoid organs, meaning that normal haematological cells will also be targeted and depleted by the ADCs. This limitation is usually compensated for by the rapid turnover capacity of the haematological compartment. As long as antigen targets are not expressed on haematopoietic stem cells (HSCs), then normal blood cells can be replenished from HSCs following temporary ADC-based depletion.

**Figure 2:**
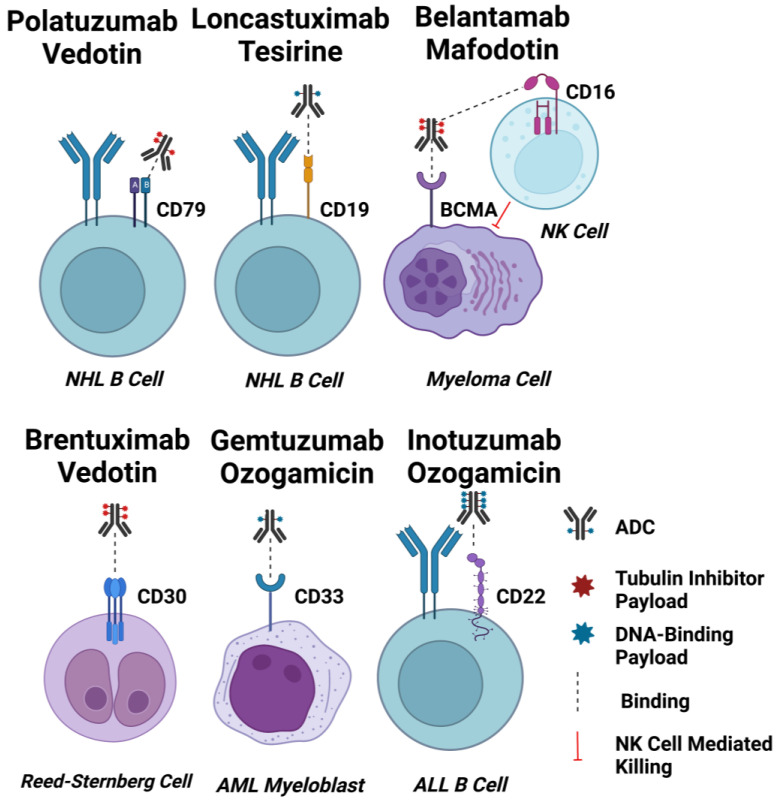
ADC Targets for Haematological Malignancies. Schematic diagrams of EMA and FDA-approved ADCs for haematological cancers binding to their target cells. Approximate drug-antibody ratios for each ADC are represented. ADC payloads that function by tubulin inhibition are shown in red, and DNA-interacting payloads are shown in blue. Dashed lines indicate binding. The effect of afucosylation on belantamab mafodotin is shown by a dashed line indicating enhanced binding to CD16 on an NK cell, and induction of NK cell-mediated killing of target cells indicated by a red line. Created using Biorender.com.

In addition to ubiquitous expression on malignant haematological cells, lack of expression on HSCs, and reduced expression or more restricted distribution on non-haematopoietic normal tissues, the target antigens in use for the approved ADCs are all readily internalised upon ligation [56]. This may be an important feature contributing to the efficacy of ADCs targeted to haematopoietic cancers. The following section describes the features of target antigens for ADCs approved for the treatment of haematological malignancies, and these are also summarised in Figure 2.

#### 2.1.1. CD33

The transmembrane glycoprotein CD33 is an adhesion molecule of the sialic acid-binding immunoglobulin-like lectin (SIGLEC) family. As a myeloid differentiation antigen, its expression on myelomonocytic-derived cells decreases upon their maturation [57]. CD33 is present on the surface of leukaemic myeloblasts in 85–90% of patients with AML, the most common acute leukaemia in adults [58]. Importantly, expression of CD33 is confined to normal and malignant myeloid cells, with no expression by non-haematopoietic lineages or HSCs [57]. It has also been reported that the average number of CD33 molecules on CD33-positive normal bone marrow cells is lower than that of bone marrow cells in acute myeloid leukaemia (AML), suggesting the potential for a therapeutic window for an anti-CD33 ADC [59,60]. Another therapeutically important characteristic of CD33 as an ADC target is its internalising property, required for the efficient delivery of cytotoxic payloads [58].

Gemtuzumab ozogamicin (Mylotarg^®^) was the first approved ADC for CD33-positive AML, consisting of a recombinant humanised anti-CD33 IgG4 kappa mAb conjugated to the DNA-cleaving calicheamicin payload via a pH-sensitive hydrazone linker [17]. This ADC is readily internalised by CD33-positive myeloid blasts, upon which hydrolysis of the hydrazone linker occurs within the relatively acidic lysosome. Subsequently, the payload causes DNA double-strand breaks, leading to cell death through apoptosis. Despite accelerated approval by the FDA in 2000, Mylotarg^®^ was voluntarily withdrawn from the market in 2010 due to poor results in a confirmatory Phase III trial. In particular, it was associated with potentially fatal hepatic veno-occlusive disease (VOD) [61,62]. Additionally, histological analyses of liver samples from patients who had experienced Mylotarg^®^-induced liver injury showed sinusoidal collagen deposition, which was attributed to the loss of CD33+ cells residing in hepatic sinusoids [63].

Myeloid cells exhibit continuously renewed expression of CD33 antigens on their cell surface upon exposure to Mylotarg^®^ [64]. This property of CD33 prompted further clinical trials (NCT00927498 and NCT00091234) using fractionated doses of Mylotarg^®^ to limit hepatic toxicity [65]. This led to re-approval by the FDA and EMA in 2017 and 2018, respectively, for combinational use with daunorubicin and cytarabine using a lower, fractionated dosing schedule for newly diagnosed CD33-positive AML adult patients and for relapsed or refractory (R/R) CD33-positive AML patients aged two years and above [66]. According to pharmacokinetic analyses, a lower maximum concentration of Mylotarg^®^ reduced the incidence of VOD without impeding CD33 saturation [65]. In 2020, the FDA further extended the indication for newly diagnosed CD33-expressing AML in paediatric patients aged 1 month and above. However, hepatotoxicity, along with infusion-related reactions and haemorrhage, remain some of the most serious adverse events (AEs) associated with the use of Mylotarg^®^ [67].

The level of CD33 expression on AML blast cells in the bone marrow has been demonstrated to positively correlate with response to treatment with Mylotarg^®^ [68]. However, high levels of CD33-positive AML blasts in peripheral blood serve as an independent negative prognostic factor for the efficacy of this agent. It has been suggested that, when there are high levels of CD33-positive AML blasts in the periphery, the administered ADC binds to and is internalised by these cells, thus depleting the supply of ADC reaching the bone marrow and the targeted CD33-positive AML blasts in this compartment [69]. Expression of CD33 has also been reported to inversely correlate with the efflux activity of multidrug resistance protein 1 (MDR1), suggesting a potential mechanism of resistance for patients with low CD33-expressing AML [70]. Overall, Mylotarg^®^ has provided robust proof of concept for CD33-targeted ADCs for AML.

#### 2.1.2. CD22

CD22 (the B-lymphoid lineage-specific transmembrane glycoprotein), a member of the SIGLEC family, is involved in the negative regulation of B-cell receptor (BCR) signalling [71]. In addition, CD22 mediates B-cell migration and maintains B cell peripheral tolerance [72]. A highly endocytic receptor with minimal shedding into the extracellular environment [73], CD22 is expressed on normal B cells, B cells in all mature B-cell acute lymphoblastic leukaemia (B-ALL), and the majority of precursor B-ALL cells [74]. However, CD22 is absent on other non-B cell lineages and HSCs, making it an attractive ADC therapeutic target for B lymphoid malignancies. Although rapid internalisation has been reported for CD22-targeted molecules, the observation of CD22 cycling between the endosomes and the plasma membrane may be a factor in reducing the effectiveness of CD22-targeted ADCs such as inotuzumab ozogamicin (Besponsa^®^) [73]. Besponsa^®^ is composed of a recombinant humanised anti-CD22 IgG4 mAb conjugated to a calicheamicin payload via an acid-labile hydrazone linker [74]. It has sub-nanomolar binding affinity and, upon binding to CD22 on the B cell surface, the ADC-CD22 complex is rapidly internalised by CD22-positive leukaemic blasts [75]. It was approved by the FDA and EMA in 2017 for the treatment of adult relapsed or refractory (R/R) B-cell precursor ALL (BCP-ALL), the most common type of adult ALL, based on the results of the Phase III INO-VATE ALL clinical trial (NCT01564784), which confirmed the superiority of Besponsa^®^ compared with standard intensive chemotherapy to serve as a bridge to haematopoietic stem cell transplantation (HCT) [76]. Notably, patients with ≥90% of CD22-positive leukaemic blasts had a longer duration of response and better overall survival compared to patients with <90% of CD22-positive leukaemic blasts in the INO-VATE study [77].

Hepatotoxicity, specifically VOD, was the most frequent Grade 3 or higher non-haematological AE [76]. The pathophysiology of VOD associated with Besponsa^®^ is not completely understood, however it has been proposed that VOD associated with both Mylotarg^®^ and Besponsa^®^ may be due to the direct effect of calicheamicin on sinusoidal endothelial cells [63]. Infection, haemorrhage, thrombocytopenia, hyperbilirubinemia and elevated transaminases are some of the other common (≥2%) AEs besides VOD [43].

In summary, as an ADC target for B-ALL, CD22, has demonstrated a favourable expression profile on B-cell malignancies and rapid internalisation characteristics. Besponsa^®^ is also effective in the treatment of R/R BCP-ALL. 

#### 2.1.3. CD19

CD19 (the B-cell transmembrane glycoprotein) is considered to be a pan-B cell marker. It has been extensively explored as a target for immunotherapies, including ADCs for B-cell non-Hodgkin lymphoma (B-NHL) [76]. Expression of CD19 is induced upon B cell lineage commitment, and expression continues from the pre-B cell stage of development until its downregulation during terminal plasma cell differentiation [78]. Alongside the complement receptor CD21, CD19 serves as a dominant signalling component of a multimolecular complex on the surface of mature B cells [79]. Importantly, CD19 expression is highly conserved in most B-cell malignancies, making it a useful diagnostic biomarker and immunotherapeutic target for B-cell-derived leukaemias and NHLs [80,81]. Additionally, CD19 possesses rapid internalisation kinetics and does not shed into the circulation, making it an ideal ADC target antigen [41]. The internalisation of anti-CD19 antibodies has been reported to inversely correlate with CD21 expression on malignant B-cell lineage cancer cells, suggesting that internalisation-dependent anti-CD19 ADCs might be less effective for CD21^high^ B-cell cancers [79]. However, this requires further evaluation in treated patient cohorts.

Loncastuximab tesirine-lpyl (Zynlonta^®^) is composed of an anti-CD19 humanised IgG1 kappa mAb conjugated to the PBD dimer SG3199, a highly cytotoxic DNA minor-groove cross-linking agent, via a protease-cleavable maleimide valine-alanine linker (together known as tesirine). Zynlonta shows an increase in targeted cytotoxicity towards CD19-positive human leukaemia and lymphoma cell lines with higher surface CD19 density [40,41]. Bystander killing of CD19-negative NHL cells by Zynlonta^®^ has also been observed in vitro [41]. In vivo, dose-dependent improvements in survival in both subcutaneously implanted and disseminated mouse xenograft models of CD19-expressing Burkitt lymphoma, ALL, and diffuse large B-cell lymphoma (DLBCL) have been reported upon administration of a single dose of this agent [41]. Finally, pharmacokinetic analysis of Zynlonta^®^ in rats and cynomolgus monkeys has demonstrated good stability and tolerability with a favourable safety profile [41].

Zynlonta^®^ was granted accelerated approval by the FDA in 2021 based on the overall response rate in the pivotal LOTIS-2 Phase II trial (NCT03589469) [40,82]. It is indicated for the treatment of adult patients with R/R large B-cell lymphoma after two or more lines of systemic chemotherapy, including DLBCL arising from low-grade lymphoma and high-grade B-cell lymphoma [40,82]. Based on the LOTIS-1 clinical trial data, Zynlonta^®^ dose adjustment and premedication with dexamethasone and spironolactone were introduced in the LOTIS-2 trial to decrease the incidence of treatment-emergent AEs related to the PBD payload. The most common Grade 3 or higher TEAEs included febrile neutropenia, thrombocytopenia and gamma-glutamyltransferase elevation. AEs with a fatal outcome were recorded in 6% of patients, but these were not considered to be associated with Zynlonta^®^ treatment and were mainly attributed to disease progression [82]. Importantly, PBD-related toxicities such as rash and oedema were reversible and generally manageable through strategic dose delays [82].

Therefore, CD19 has proven to be a viable ADC target antigen with favourable properties including differential expression on malignant versus normal tissues and a low rate of shedding, as demonstrated by the efficacy and acceptable toxicity profile of Zynlonta^®^.

#### 2.1.4. CD79b

CD79 (a B-cell receptor transmembrane protein) is restricted to B cells and highly expressed by most B-NHLs [83]. Two immunoglobulin chains, CD79a and CD79b, comprise the heterodimer CD79, a component of the BCR signalling complex [84]. Following antigen recognition by the BCR, CD79 is efficiently internalised and trafficked to the lysosome-like compartment as part of its function in MHC (the major histocompatibility complex) Class II-mediated antigen presentation [84,85]. Therefore, CD79a and CD79b are promising targets in the treatment of B-NHLs for internalising ADCs with stable linkers which require complete lysosomal degradation for payload release [84]. CD79b has been demonstrated to be a more effective ADC target in comparison to CD79a [84]. For example, a study by Polson et al. showed that a single dose of anti-CD79b ADCs (with MMAF or DM-1 payloads) provided sustained tumour regression or complete remission in tumour-bearing mice, whereas tumours recurred in mice treated with equivalent anti-CD79a ADCs [84].

The CD79b-targeted ADC polatuzumab vedotin-piiq (Polivy^®^) is composed of a recombinant humanised IgG1 mAb conjugated to the anti-mitotic payload MMAE. Upon administration to patients, Polivy^®^ selectively binds to CD79b on the surface of B-NHL cells and is then readily internalised via BCR cross-linking and endocytosis for intracellular delivery of the MMAE, which impedes microtubule polymerisation and leads to apoptosis of CD79-positive malignant B cells [86]. In vitro studies of Polivy have demonstrated cytotoxicity in the majority of activated B-cell-like (ABC) and germinal centre B-cell-like (GCB) DLBCL cell lines. Responses were observed irrespective of CD79b mutations, which predominantly occur in ABC-DLBCL, and are associated with poorer survival outcomes [87]. Responses to Polivy^®^ were also observed in both DLBCL subtypes in the Phase I clinical trials NCT01290549 [87]. Notably, in vivo data from Pfeifer et al. showed that the response of DLBCL patients to Polivy did not correlate with CD79b expression levels as detected by immunohistochemistry (IHC), suggesting that patients with low CD79b expression levels on their DLBCL cells (as detected by IHC) may also potentially benefit from Polivy^®^ treatment [87].

Polivy^®^ was granted accelerated approval by the FDA and EMA in 2019 and 2020, respectively, for use in combination with the chemotherapeutic agent bendamustine and the anti-CD20 monoclonal antibody rituximab. This combination therapy (known as Pola-BR) is approved for adult patients with R/R DLBCL who have received at least two prior therapies in the US or one line of treatment in Europe [86]. R-CHOP (a combination of rituximab, cyclophosphamide, doxorubicin, vincristine and prednisone) is the standard first-line treatment for DLBCL [88], and the approval of Pola-BR was based on a superior complete response rate and an improved duration of response compared to the bendamustine/rituximab combination alone (Study GO29365; NCT02257567) [89].

Modest systemic release of MMAE and the clearance of intact Polivy or its metabolites are assumed to be responsible for the off-target adverse events such as medullary toxicity and peripheral neuropathy, two of the most common but manageable side effects. In 64% of patients, serious AEs occurred in Pola-BR-treated patients, mostly involving infections. Also, 18% of patients had to discontinue treatment due to AEs including cytopenias [90]. The realization that systemic linker cleavage in ADCs such as Polivy^®^ may lead to payload-related AEs has led ADC researchers to optimise linker technologies to improve the safety profiles of next generation ADC products [91].

Overall, the suitability of CD79b as an ADC target antigen has been validated by the superior efficacy of Pola-BR compared with the bendamustine/rituximab combination alone for the treatment of DLBCL. However, the lack of correlation between CD79b expression (as measured by IHC) and the clinical response to Polivy^®^ [87] suggests that further research is required to elucidate suitable biomarkers to predict the response to Polivy^®^. One potential prognostic tool is the level of circulating tumour DNA (ctDNA) in the periphery, with high baseline ctDNA being an indicator of a high risk of lymphoma progression [92].

#### 2.1.5. BCMA

BCMA (the transmembrane glycoprotein B-cell maturation antigen) is a member of the TNFR (tumour necrosis factor receptor) superfamily that is selectively induced during plasma cell differentiation [93]. Upon binding to its B-cell activating factor (BAFF) and a proliferation-inducing ligand (APRIL), BCMA supports the survival of plasmablasts and bone marrow plasma cells [94]. The same signalling pathway has been shown to support myeloma cell growth and survival [94]. Because of its universally high expression levels on normal plasma cells and multiple myeloma (MM) cells, and a lack of expression on naïve and memory B cells, BCMA appears to be a promising target for the treatment of MM.

The BCMA-targeted ADC belantamab mafodotin-blmf (Blenrep^®^) is indicated for heavily pre-treated adult patients with progressing R/R MM who have received at least four prior therapies, including an anti-CD38 mAb, a proteasome inhibitor, and an immunomodulatory agent [95]. It was the first approved anti-BCMA therapy, receiving accelerated FDA approval as a monotherapy in 2020 and EMA approval the same year. These approvals were based on results from the pivotal DREAMM-2 clinical study (NCT03525678), which was contingent upon confirmed benefits from a randomised phase III clinical trial [34]. 

Blenrep^®^ comprises a humanised, Fc-enhanced, engineered afucosylated IgG1 mAb conjugated to MMAF via a non-cleavable maleimidocaproyl linker [95]. Use of the charged membrane-impermeable MMAF payload restricts MMAF-mediated tumour lysis in BCMA-positive cells [93]. Afucosylation of the anti-BCMA mAb significantly increases the affinity of the antibody Fc to the FcγR expressed by NK cells and macrophages, thereby increasing the infiltration of NK cells and macrophages, and thereby facilitating antibody-dependent cell-mediated phagocytosis [93].

The most common side effect of Blenrep^®^, keratopathy, occurs in approximately 70% of patients. This ocular AE is attributed to off-target damage to the corneal epithelium by the MMAF payload. However, it may be reduced by ADC dose modifications, although it still requires close monitoring by healthcare professionals, including eye specialists [96]. Studies have been conducted to investigate the patient impact and corneal changes produced by ADC-induced keratopathy [97,98].

In November 2022, GSK announced that it had initiated the withdrawal of the US marketing authorisation for Blenrep^®^ following a request from the FDA because the confirmatory DREAMM-3 trial (NCT0416221) had not met its primary end point of progression-free survival (PFS) [99].

#### 2.1.6. CD30

CD30 (a transmembrane glycoprotein of the tumour necrosis factor receptor (TNFR) is part of a superfamily expressed on a small subset of activated B- and T-cells, and on various lymphoid neoplasms [100,101]. Binding of CD30 to its cognate CD30 ligand (CD30L) results in recruitment of the TNFR-associated factor (TRAF) and TRAF-binding proteins 1, 2, and 5. This leads to increased survival and proliferation of neoplastic cells, mediated through NF-kB and kinase signalling pathways [100,102]. The abundant expression of CD30 in Hodgkin Reed-Sternberg cells of classical Hodgkin lymphoma (cHL), anaplastic large cell lymphoma (ALCL) and cutaneous T-cell lymphoma (CTCL), combined with relatively restricted CD30 expression by normal tissues, means that anti-CD30 ADCs can selectively target CD30-positive lymphoid tumours [13,100,101]. 

Brentuximab vedotin (Adcetris^®^) is an FDA- and EMA-approved ADC consisting of a humanised CD30-targeted IgG1 mAb conjugated to MMAE via a cathepsin B-sensitive valine-citrulline linker [103]. After binding to CD30 on the surface of lymphoma cells, Adcetris^®^ is readily internalised and then trafficked to lysosomes, where the linker is cleaved by proteases followed by release of the MMAE. Currently, Adcetris^®^ is approved as a front-line treatment for Stage III/IV classical HL in combination with chemotherapy (typically doxorubicin, vinblastine and dacarbazine) [104] and for the treatment of R/R NHLs, including primary ALCL, CTCL and CD30-positive mycosis fungoides [102].

Dose-limiting peripheral sensory and motor neuropathy is frequently experienced by patients receiving Adcetris^®^, leading to dose modification or treatment discontinuation. Adcetris^®^-induced axon degeneration and peripheral neurotoxicity are thought to be caused by off-target effects of the microtubule-binding MMAE payload, and not related to the CD30 target antigen [105]. Due to the long projections involved, axonal transport between neuronal cell bodies and distal nerve endings is highly microtubule-dependent, making peripheral nerves especially susceptible to MMAE toxicity [106].

In summary, CD30 is effectively targeted by Adcetris^®^ for the treatment of several lymphomas, thus validating it as an ADC target. The neuronal toxicities associated with the MMAE payload can be serious but are manageable by modifications to the dosing regimens.

### 2.2. ADC Targets for Solid Tumours

**Figure 3:**
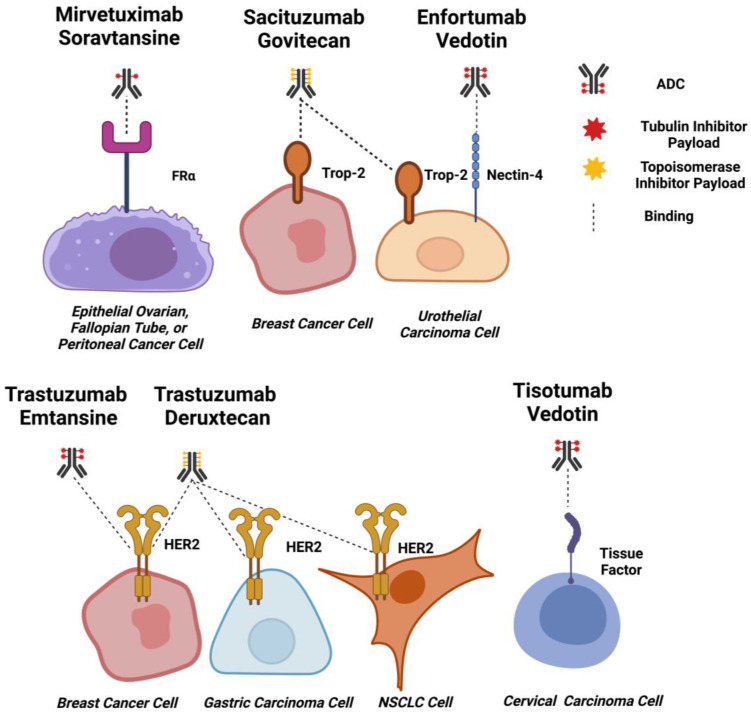
ADC Targets for Solid Tumours. Schematic diagrams of EMA and FDA-approved ADCs for solid tumours binding to target cells. Approximate drug-antibody ratios for each ADC are represented. ADC payloads that function by tubulin inhibition are shown in red, and topoisomerase inhibitors are shown in yellow. Dashed lines indicate binding. Created using Biorender.com.

ADCs designed to treat solid tumours are directed against a range of antigens, which generally comprise tumour-associated membrane glycoproteins or receptors that may be implicated in pro-tumourigenic pathways [12]. ADCs approved to date by the FDA and EMA for the treatment of solid tumours target HER2 (Human Epidermal Growth Factor Receptor 2), TROP2 (Trophoblast Antigen 2), Nectin-4, FRα (Folate Receptor Alpha) and TF (Tissue Factor) (Figure 3). These are discussed in detail in the sections below.

#### 2.2.1. HER2

HER2 (the Human Epidermal Growth Factor Receptor 2) is a transmembrane receptor whose over-expression has been reported in several malignancies, including biliary tract, colorectal, non-small cell lung (NSCLC), bladder and breast cancers [107]. Over-expression of HER2 is reported in 10–20% of breast cancers and is associated with reduced disease-free survival (DFS) and overall survival (OS) [108,109]. HER2-positive breast cancers are classified by the immunohistochemical staining of HER2 in at least 10% of cells or by the presence of HER2 gene amplification [108]. This subset of breast cancers exhibits increased HER2 signalling, inducing mitogen-activated protein kinase cascades that potentiate the up-regulation of proliferative and pro-survival genes that contribute to cancer survival and progression [108].

Expression of HER2 on normal haematopoietic cells and other cell types has been reported at both the mRNA and protein levels, but at lower levels compared to malignant cells [110,111,112]. This suggested that there could be a therapeutic window for HER2-targeted therapies and led to the development of targeted single antibodies and ADCs for the treatment of HER2-positive breast cancer. A potential challenge for the development of anti-HER2 ADC therapeutics is the reported shedding of the HER2 antigen [113,114], and this is addressed in greater detail later in the review.

At present, there are two approved ADCs targeting HER2 for the treatment of HER2-positive breast cancer, both based on the anti-HER2 IgG1 antibody trastuzumab (Herceptin^®^) conjugated to either the tubulin-inhibiting mertansine (i.e., trastuzumab deruxtecan; T-Dxd or Enhertu^®^), or the topoisomerase-inhibiting deruxtecan (i.e., trastuzumab emtansine; T-DM1 or Kadcyla^®^). The features of Kadcyla^®^ and Enhertu^®^ are compared in Table 3.

Studies of trastuzumab, approved by the FDA in 1998 for the treatment of HER2-positive breast cancer [115], revealed some challenges for targeting HER2 with antibody-based therapeutics. In vitro observations showed that upon binding, trastuzumab-HER2 complexes are internalised but may be rapidly recycled to the plasma membrane, a process that has since been established as a resistance mechanism for anti-HER2 antibody therapies [116,117,118]. Other studies revealed that trastuzumab can cause cardiac toxicity due to the presence of HER2-expressing cardiomyocytes, which led to the development of exclusion criteria and rigorous monitoring during the clinical trials of trastuzumab and trastuzumab-based therapeutics [119]. Trastuzumab is postulated to block the binding of neuregulin to HER2 expressed on cardiomyocytes, thus inhibiting the potentiation of signalling cascades that induce protective mechanisms against oxidative stress [120,121,122]. Risk factors for trastuzumab-mediated cardiotoxicity include factors such as age, previous anthracycline exposure and history of cardiac dysfunction. A key indicator of a propensity for trastuzumab-mediated cardiotoxicity is low left ventricular ejection fraction (LVEF), which is a marker of the contractile function of the heart [120].

Knowledge of trastuzumab-mediated toxicity has informed the design of clinical studies evaluating Kadcyla^®^ and Enhertu^®^. In a Phase II trial evaluating the cardiac safety and efficacy of Kadcyla in patients with early-stage HER2-positive breast cancer pre-treated with anthracycline-based chemotherapy, exclusion criteria included cardiotoxicity from the prior chemotherapy treatment or any other cardiac issues. In this study, complete responses were observed in 56% of patients, with only 3.4% of individuals experiencing cardiac AEs related to Kadcyla^®^ and with mean LVEF remaining stable in the treated group [123]. Pooled analysis of Kadcyla^®^-mediated cardiotoxicity available in 2020 and based on data from seven clinical trials revealed that in HER2-positive advanced breast cancer, the most common cardiac event was a low-grade reduction in LVEF which, for the 79% of patients experiencing this effect, returned to near-normal levels within one year [124].

Phase III trials of Kadcyla^®^ provided further safety and efficacy data. The Phase III EMILIA trial, the results of which formed the basis of the FDA approval of Kadcyla ^®^, evaluated Kadcyla against capecitabine in combination with the dual tyrosine kinase inhibitor lapatinib, which targets EGFR and HER2, in patients with HER2-positive breast cancer previously treated with trastuzumab and taxane. Significant increases in OS (overall survival) and PFS were observed in the Kadcyla^®^ group compared to the capecitabine/lapatinib group. More frequent Grade 3 and above AEs were observed in the chemotherapy plus targeted therapy (control) arm than in the arm treated with Kadcyla^®^ [125,126]. A similar distribution of Grade 3 or higher AEs in favour of the ADC was also observed in the Phase III TH3RESA trial evaluating Kadcyla^®^ against the physician’s choice of chemotherapy for previously treated HER2-positive breast cancer, as well as in the Phase III MARIANNE study evaluating Kadcyla^®^ as a monotherapy or in combination with the anti-HER2 IgG1 humanised monoclonal antibody pertuzumab against a combination therapy of trastuzumab and taxane [127]. These studies provided further evidence that stringent inclusion criteria can reduce the incidence of trastuzumab-mediated cardiotoxicity, and identified the main Kadcyla^®^-associated toxicities as thrombocytopenia, anaemia and raised levels of aspartate aminotransferase [125,126,127].

Enhertu^®^ differs from Kadcyla^®^ in that its payload is a topoisomerase inhibitor rather than a tubulin inhibitor. It also has a higher DAR (Drug-Antibody Ratio) of 7.7 compared to 3.5 for Kadcyla^®^. Enhertu^®^ was approved by the FDA for the treatment of HER2-positive metastatic breast cancer based on the results of the Phase II Destiny Breast 01 trial, in which responses were observed in 60.9% of patients [128], and the Phase Ib trial DS8201-A-J101, which provided evidence of safety in patients with HER2-low breast cancer [129]. Commonly observed toxicities were neutropenia, anaemia, nausea and interstitial lung disease [128,129].

When considering the threshold of target antigen expression required for ADC efficacy, Enhertu^®^ has recently provided useful results in patients with HER2-low malignancies. The presently ongoing Phase II Destiny Breast 04 trial is evaluating the effectiveness of Enhertu^®^ for the treatment of HER2-low unresectable or metastatic breast cancer compared with chemotherapy (i.e., physician’s choice of capecitabine, eribulin, gemcitabine, paclitaxel or nab-paclitaxel). Interim results have shown that Enhertu^®^ significantly increased PFS, the primary endpoint, compared to chemotherapy [130]. Despite the exclusion of patients with a history of interstitial lung disease from this trial, a similar percentage of Enhertu^®^-treated patients have developed interstitial lung disease (12.1%), comparable with the 13.6% observed in the Destiny Breast 01 trial. However, in both trials, most of these toxicities were either Grade 1 or 2 [129,130].

Kadcyla^®^ utilises a non-cleavable maleimidomethyl cyclohexane-1-carboxylate (MCC) linker to join trastuzumab to approximately 3.5 molecules of DM1 per antibody, whereas Enhertu^®^ uses a cleavable tetrapeptide linker and deruxtecan at a DAR of approximately 8.0 [117,131]. Therefore, a comparison of Kadcyla^®^ and Enhertu^®^ provides an opportunity to study how ADCs targeted to the same antigen can produce different efficacy and toxicity profiles, presumably relating to the different linker and payload types and DARs. This is being studied in the Phase III Destiny Breast 03 trial, which randomly assigns Kadcyla^®^ or Enhertu^®^ to patients with HER2+ unresectable or metastatic breast cancer who progressed after treatment with trastuzumab and taxanes [132]. Interim analysis has revealed that after 12 months, PFS was 25.1 months in the Enhertu^®^-treated patients compared to 7.2 months for the Kadcyla^®^ cohort, with OS being 79.7% and 34.2%, respectively. However, the superior efficacy of Enhertu^®^ was associated with a marginally greater toxicity compared to Kadcyla^®^. Drug-related AEs were 98.1% for Enhertu^®^ compared with 86.6% for Kadcyla^®^, with 10.5% of Enhertu-treated patients experiencing pneumonitis or interstitial lung disease. Low rates of cardiotoxicity were observed in both the Enhertu^®^ and Kadcyla^®^ arms, with decreases in LVEF observed in 0.4% and 2.3% of patients, respectively [132]. The effectiveness of Enhertu^®^ in advanced pre-treated breast cancer in these studies led to expansion of the initial 2019 FDA approval of this agent. Although it was originally approved for patients with unresectable or metastatic HER2-positive breast cancer who had previously been treated with two or more anti-HER2 therapies in a metastatic setting, following the Phase III Destiny Breast 03 trial, Enhertu^®^ was also approved for patients who had received prior anti-HER2 therapies in metastatic, neoadjuvant or adjuvant settings, and had experienced recurrence during therapy or within six months after initial treatment [132,133].

In addition to HER2-positive breast cancer, Enhertu^®^ is also approved by the FDA for the treatment of patients with HER2-mutated non-small-cell lung carcinoma (NSCLC) who have received at least one prior systemic therapy [134] based on the results of the Destiny Lung-02 trial [135]. It is also approved for the treatment of patients with HER2-positive, locally advanced gastric or gastroesophageal junction cancers previously treated with a trastuzumab-based therapy [136]. The treatment of other HER2-expressing cancer types with ADCs such as Enhertu^®^ is likely to be explored in the future. 

In conclusion, Kadcyla^®^ and Enhertu^®^ comprise safe and effective ADCs targeting HER2 for the treatment of breast and, in the case of Enhertu^®^, lung and gastric cancers. The success of these two ADCs is partly based on early clinical experience with trastuzumab in excluding patient groups in which AEs such as cardiotoxicity are most likely to be severe.

#### 2.2.2. TROP-2

TROP-2 (Trophoblast Antigen 2) is a membrane glycoprotein, the expression of which has been reported in several carcinoma types as well as on the epithelia of many healthy tissues, both transcriptionally and at the protein level. For example, expression is found on tissues such as the skin and the epithelia of the uterine cervix, tonsils and thymus [137,138]. TROP-2 participates in cellular signalling through the extracellular binding of IGF-1 (Insulin-Like Growth Factor 1) or MDK (Midkine), which stimulates pro-survival and cell proliferation pathways [139].

Proteolytic cleavage of TROP-2 into extracellular and intracellular domains has been identified as a probable mechanism for how the protein acts as a driver of progression in prostate cancer, with the intracellular domain translocating to the nucleus to interact with mediators of cell growth and proliferation [140]. In vivo studies have suggested that proteolytic cleavage at the ADAM10 cleavage site R87/T88 is essential for the role of TROP-2 in cancer growth and metastasis. Experiments have shown that transfection of cells with TROP-2 containing mutated residues at these sites can restrict the growth of fibrosarcoma and of transformed human embryonic kidney cell line xenografts, and reduce the volume of liver metastases of a colorectal cancer cell line injected into athymic nude mice [141]. In addition, in vitro studies have indicated that when fully glycosylated, internalised TROP-2 may be directed to endocytic processing pathways or recycled to the plasma membrane [142], suggesting that the effectiveness of a TROP-2-targeting ADC that binds to fully glycosylated TROP-2 may be impeded by direction of the ADC-antigen complex to a recycling pathway.

Immunohistochemical analysis of large cohorts of breast cancer patients has established that the presence of membrane-associated TROP-2 is significantly associated with a poorer prognosis [142]. Furthermore, studies of TROP-2 expression in TNBC patient cohorts have shown that, in each cohort, more than 80% of patients exhibited moderate to strong membrane expression of TROP-2 in 10% or more of cells from TNBC tumours as assessed by IHC [143]. Overall, many studies have now confirmed that TROP-2 is a mediator of pro-tumourigenic signalling in a number of malignancies, including TNBC, in which its expression is now recognised as a prognostic marker. 

The ADC sacituzumab govitecan (Trodelvy^®^) comprises an anti-TROP-2 IgG1 antibody conjugated to SN-38, a topoisomerase inhibitor, through a cleavable linker. It is approved by the FDA for the treatment of relapsed or metastatic triple-negative breast cancer (TNBC) and for refractory metastatic urothelial cancer [144]. The first approval of Trodelvy^®^ for TNBC was based on the results of the Phase I/II IMMU-132-01 trial, in which an overall response rate of 33.3% and a medium duration of response of 7.7% were observed. Common toxicities included nausea, neutropenia, fatigue and diarrhoea [145]. These results led to the accelerated approval of Trodelvy^®^ for TNBC by the FDA in 2020.

Regular approval was subsequently granted in 2021 based on the results of the Phase III ASCENT trial [144,146] which evaluated Trodelvy^®^ compared with chemotherapy in patients with relapsed or refractory triple-negative breast cancer based on primary endpoints including progression-free survival. In patients without brain metastases, median PFS was 5.6 months for Trodelvy^®^ compared with 1.7 months for chemotherapy, and median OS was 12.1 and 6.7 months, respectively. Including patients with brain metastases, PFS and OS were 4.8 and 11.8 months, respectively, for Trodelvy^®^-treated groups, compared with 1.7 months and 6.9 months for chemotherapy-treated cohorts [147]. Interestingly, compared to conventional chemotherapy, Trodelvy^®^ resulted in toxicities in a higher proportion of patients, with the most common Grade 3 or above AEs including neutropenia, leukopenia and diarrhoea [147]. Although there has been criticism of the study design of the ASCENT trial [148], the quality of the data from the trial was bolstered by biomarker analysis, which revealed benefits for patients whose tumours were expressing moderate levels of TROP-2 [149], thus suggesting that high levels of TROP-2 expression by malignant cells might not be necessary for Trodelvy^®^ to benefit patients.

In addition to its approval for the treatment of metastatic TNBC, in 2023 the FDA approved Trodelvy^®^ for the treatment of patients with locally advanced, hormone receptor (HR)-positive, HER2-negative breast cancer who had received at least one endocrine therapy and two or more systemic therapies [150]. This approval was based on the results of the TROPiCS-02 Phase III study which evaluated Trodelvy^®^ for the treatment of patients with pre-treated, unresectable or locally advanced metastatic HR-positive, HER2-negative breast cancer against single-agent chemotherapy [151]. PFS in the Trodelvy^®^-treated arm was 5.5 months compared to 4 months in the chemotherapy-treated arm, with a greater number of TEAEs (commonly neutropenia and diarrhoea) occurring in the chemotherapy-treated arm [151].

As well as for breast cancer, Trodelvy^®^ is also approved by the FDA for the treatment of urothelial carcinoma. Accelerated approval for urothelial cancer was granted in 2021 based on results from the Phase II TROPHY-U-01 trial which evaluated the effectiveness of the ADC in locally advanced or metastatic urothelial carcinoma that had progressed following platinum-based chemotherapy or checkpoint inhibitor therapies [152]. Overall response rates were found to be 27.4% and 5.3% for the Trodelvy^®^-treated patients versus the control arm, respectively. The most common Grade 3 and above toxicities for Trodelvy^®^ observed in >5% of patients included neutropenia, leukopenia, anaemia and diarrhoea, most of which could be managed by dose delay or interruption [152].

Therefore, TROP-2 has been demonstrated to be a viable ADC target antigen for the treatment of TNBC and urothelial carcinoma, despite its reported expression in some normal tissues and evidence of recycling to the cell surface.

#### 2.2.3. Nectin-4

Nectin-4 (Poliovirus Receptor-Related Protein 4) is a Type I transmembrane polypeptide member of the nectin family of immunoglobulin-like adhesion molecules [153]. It is thought to mediate calcium-independent cell-cell adhesion at the cytoskeletal complexes, known as adherens junctions, by modulating cytoskeleton rearrangements [153]. In normal tissues, Nectin-4 is mainly expressed in the embryo and placenta while expressed at only a low level in healthy adult tissues [154]. Nectin-4 is reported to be overexpressed in various epithelial cancers. Immunohistochemical (IHC) analysis of 2394 patient biopsies demonstrated that 69% of cancers of epithelial origin were positive for Nectin-4, with strong staining observed in 60% of bladder tumours and 53% of breast cancer samples (H-score ≥ 100). Metastatic bladder cancer biopsies also gave a similar H-score for Nectin-4 expression compared with their primary tumours [155]. In contrast, homogenous weak to moderate staining was observed across a panel of thirty-six normal tissues. These findings suggested a potentially favourable therapeutic window for the development of an antibody or ADC therapeutic targeted to Nectin-4 [155].

In patients with metastatic breast cancer, Nectin-4 shedding has been reported to be constitutive. Soluble Nectin-4 is formed by the entire Nectin-4 ectodomain and is produced by proteolytic cleavage at the cell surface by metalloproteinases [156]. Expression of Nectin-4 or ADAM10 and ADAM17, the proteases that cleave it, has been reported to inversely correlate with PFS in patients with Grade 1 or 2 serous ovarian cancer [157], further supporting the potential of Nectin-4 as a therapeutic target. Studies have yet to fully elucidate the precise internalisation mechanisms and cycling properties of Nectin-4.

Enfortumab vedotin (Padcev^®^) is an ADC comprising the human anti-Nectin-4 IgG1 kappa mAb conjugated to MMAE via a proprietary protease-cleavable linker. During pre-clinical development, Padcev^®^ demonstrated antigen target specificity and anti-tumour efficacy in bladder and breast cancer patient-derived xenografts (PDXs) in vivo. In contrast, the unconjugated anti-Nectin-4 antibody failed to induce cytotoxicity in vitro or to have anti-tumour activity in vivo in xenograft models based on relevant target-expressing cell lines, suggesting that delivery of a potent cytotoxic payload such as MMAE may be critical for successful therapeutic exploitation of the Nectin-4 antigen [155].

Following accelerated approval in 2019, in 2021 the FDA gave full approval to Padcev^®^ for the treatment of adult patients with locally advanced or metastatic urothelial carcinoma whose disease had progressed on a PD-1 (Programmed Death Receptor-1) or PD-L1 (Programmed Death-Ligand 1) inhibitor and a platinum-based chemotherapy such as cisplatin. In 2020, Padcev^®^ was also granted breakthrough therapy designation by the FDA as a first-line treatment in combination with the checkpoint inhibitor pembrolizumab for cisplatin-ineligible patients with locally advanced or metastatic urothelial carcinoma [158].

TEAEs associated with Padcev^®^ include rash and peripheral neuropathy, with the latter being the most common and resulting in dose reduction and/or treatment discontinuation. The development of a rash is a predictable on-target off-tumour toxicity for Padcev^®^ given the moderate expression of Nectin-4 in normal human skin [155]. An association of peripheral neuropathy with MMAE due to its effects on the neuronal cell body is well documented and predictable [159].

In conclusion, enfortumab vedotin (Padcev^®^) has validated Nectin-4 as an ADC target for the treatment of patients with advanced or metastatic urothelial carcinoma who are refractory to PD-1 or PD-L1-based immunotherapy or platinum-based chemotherapy. Furthermore, the specific toxicities of this ADC can be attributed to off-target expression of Nectin-4 and the systemic toxicity of its tubulin-inhibiting payload.

#### 2.2.4. Tissue Factor

TF (Tissue Factor), also known as Thromboplastin Factor III or CD142, is a transmembrane glycoprotein with pro-coagulant activity and the capacity to induce intracellular signalling in complex with the proteolytic enzyme Factor VIIa (FVIIa) [160]. TF is thought to contribute to cancer progression via FVIIa-dependent intracellular signalling pathways that regulate cell survival, proliferation, metastasis and angiogenesis. It is frequently up-regulated in various solid tumours and the tumour vasculature as a result of hypoxia-induced signalling. Specifically, TF is highly prevalent in cervical cancer and has been associated with a poor prognosis [161]. It is widely expressed in various organs, although expression is mostly restricted to cells of the subendothelial vessel wall [162].

The internalizing property of this antigen is ideal for the development of TF-targeted ADCs. Of further interest is the reported mechanism of TF-FVIIa-mediated induction of surface TF expression, in which binding of FVIIa to TF and formation of the TF-FVIIa complex leads to release of TF from the Golgi apparatus followed by trafficking to the membrane resulting in enhanced cell surface TF expression [163]. If this effect can be induced by an anti-TF ADC, then this may allow repeated targeting of TF-expressing malignant cells.

Tisotumab vedotin (Tivdak^®^) is an anti-TF ADC comprising a TF-targeted fully human monoclonal IgG1 antibody conjugated to MMAE via a protease-cleavable valine-citrulline linker. The high potency of Tivdak^®^ in heterogenous tumours may be due to the bystander effect of the MMAE payload once released, diffusing across the membranes of neighboring cells with reduced or no TF expression.

Based on the InnovaTV 204 clinical trial (NCT03438396), in 2021 the FDA granted accelerated approval for the treatment of patients with recurrent or metastatic cervical cancer who had received no more than two prior systemic therapies. In the clinical trials, significant TEAEs included peripheral sensorimotor neuropathy and pyrexia [164], the former being attributed to the MMAE payload. Ocular toxicity, known to be associated with MMAE, was also a common side effect and led to dosage reductions and/or treatment discontinuation [165]. Other AEs included haemorrhaging and severe inflammation of the lungs.

In conclusion, Tivdak^®^ is an effective therapeutic agent for the treatment of TF-expressing metastatic cervical cancer, and so provides validation for this ADC antigen. 

#### 2.2.5. FRα

FRα (Folate Receptor Alpha) is a membrane-bound metabolic folic acid receptor involved in the intracellular trafficking of folic acid. Once bound to folic acid, the receptor-ligand complex internalises through a non-classical lipid raft endocytic mechanism that involves membrane invagination, trafficking to the endosomes, endosomal-lysosomal fusion and then acidification before release of folate into the intracellular environment [166,167]. The direction of receptor-ligand complexes to an acidified cellular compartment, combined with the reported high expression of FRα in ovarian, breast and lung cancer subsets compared to restricted expression in normal cells at the mRNA and protein levels, makes the receptor ideal for targeting with an ADC [166,168]. FRα is postulated to aid pro-tumourigenic signalling through binding to folate, inducing downstream effects such as activation of STAT3, intracellular transport of FRα to act as a transcription factor for pro-growth pathways, and intracellular transport of folic acid for DNA biosynthesis [166].

The effectiveness of an ADC targeting FRα may be impacted by the reported shedding of FRα into the periphery, which is observed in healthy subjects but enhanced in patients with ovarian cancers that over-express FRα [168]. However, an in vitro study using the anti-FRα IgE antibody MOv18 IgE revealed that recombinant FRα at levels similar to those observed in ovarian cancer patients in vivo did not reduce levels of human peripheral blood mononuclear cell (PBMC)-mediated MOv18 IgE-induced antibody-dependent cellular cytotoxicity (ADCC) against a FRα-positive ovarian cancer cell line. Recombinant FRα only impeded MOv18 IgE-mediated ADCC at FRα concentrations equivalent to serum levels observed in the top 8% of patient samples. Crucially, blockade of MOv18 IgE-induced ADCC of ovarian cancer cells by PBMCs was partially surmounted by increasing the concentration of the antibody, MOv18 IgE [168]. Therefore, these results suggest that any impeding effects that soluble FRα may expert upon ADC binding to FRα on the surface of malignant cells may be circumvented by increasing the therapeutic dose of an antibody and by extending the use of an ADC.

An ADC that targets FRα (mirvetuximab soravtansine; Elahere^®^) has received accelerated approval by the FDA for the treatment of epithelial ovarian cancers. Elahere consists of a humanised anti-FRα IgG1 antibody conjugated to the tubulin-inhibiting payload DM-4 through a glutathione-cleavable linker [169]. Pre-clinical studies of this ADC found that it produced bystander killing effects and restricted the growth of human tumours in xenograft mouse models of FRα-expressing ovarian cancer and NSCLC [169].

Clinical evaluations of Elahere^®^ yielded divergent results depending on the selection of patient groups. For example, the FORWARD I Phase III trial evaluated the effectiveness of Elahere^®^ against chemotherapy (including paclitaxel, doxorubicin and topotecan) for the treatment of patients with pre-treated, platinum-resistant epithelial ovarian cancers that were FRα-positive by IHC. In this trial, differences in PFS between the Elahere^®^ and chemotherapy-treated arms were not found to be statistically significant, although overall response rates, toxicity profiles and patient-reported outcomes were more favourable in the Elahere^®^-treated arms than for chemotherapy [170]. The selection of patients with tumours expressing high levels of FRα by IHC has led to more favourable results in subsequent Phase III clinical trials. For example, results from the SORAYA Phase III trial evaluating Elahere^®^ in patients with platinum-resistant ovarian cancer pre-treated with one to three prior lines of systemic therapy, and with high tumour expression of FRα, revealed an overall response to Elahere^®^ of 32.4%. Most importantly, five complete responses out of a total of 106 patients were observed [171]. Commonly observed Elahere^®^-related AEs observed in both trials included nausea, diarrhoea and fatigue, as well as blurred vision and keratopathy, which were largely managed with dose delays and/or reductions [170,171].

Based on the results of the SORAYA trial, in 2022 the FDA granted accelerated approval to Elahere^®^ for the treatment of FRα-positive, platinum-resistant epithelial ovarian cancer that has previously been treated with one to three prior systemic treatments [172].

Based on these results, it is evident that FRα is a validated ADC antigen, although appropriate levels of FRα expression are required to evoke clinical benefit, making patient selection important.

## 3. Target Selection in the Design of ADCs

### 3.1. Target Expression Levels

Antigen expression threshold levels sufficient for ADC activity vary significantly depending on several parameters, many of which are yet to be fully elucidated. However, expression levels are known to be dependent on the specific target, the antigenic epitope recognised and the cancer indication, either individually or in combination [13]. This is especially evident for ADCs targeted to solid tumours. For example, clinical experience in evaluating the effectiveness of Kadcyla^®^ against HER2-positive metastatic breast cancer has demonstrated better survival outcomes in high compared with low HER2-expressing subgroups [44,173].

Interestingly, the effectiveness of Enhertu^®^ in breast cancer patients with low HER2 expression levels highlights the fact that there is no widely applicable threshold of sufficient expression on target tissues to ensure the efficacy of an ADC [130]. Thus, HER2-ultra-low cancers, a categorization that accounts for less than ten percent of breast cancers that exhibit IHC staining scores of zero while still showing faint staining for HER2, are now being considered for treatment with anti-HER2 therapies [174]. This is bolstered by the observation that some HER2-negative cell lines may still maintain active HER2 signalling and are susceptible to anti-HER2 therapies in both in vitro and in vivo experiments. This suggests that low levels of pro-tumourigenic signalling due to low levels of surface-expressed HER2 may still support tumour growth, and that abrogation of this tumour-supporting pathway through anti-HER2 therapies can restrict tumour growth and promote survival [175]. This is somewhat at odds with the recent clinical evaluation of Elahere^®^, in which the selection of patient groups with only high expression of the FRα target antigen appears to be associated with therapeutic benefit [170,171]. Furthermore, in the case of CD70, an ADC target under clinical investigation for renal cell carcinoma, only a limited correlation between antigen expression levels and sensitivity to CD70-targeted ADCs was observed [176]. 

Overall, preclinical studies and clinical evaluations of ADCs for the treatment of several cancer types have shown that no overarching paradigm exists for correlating antigen expression level with ADC activity. Thus, the desirable cut-off value of antigen expression needs to be empirically determined for each tumour type and ADC [177].

In this context, it is interesting to note that, of the twelve currently FDA-approved ADCs, only three (Kadcyla^®^, Enhertu^®^ and Elahere^®^) have a formal requirement in their licensing for an antigen test (e.g., the HER2 HercepTest^®^, Ventana PATHWAY anti-HER-2/neu (4B5) rabbit monoclonal primary antibody assay, the INFORM HER2 Dual ISH DNA Probe Cocktail assay for Kadcyla^®^, and the FRα Ventana FOLR-2.1 RxDx companion diagnostic test for Elahere^®^) prior to the commencement of treatment [172,178,179]. This may, in part, relate to the fact that Kadcyla^®^ and Enhertu^®^ are based on trastuzumab, for which there is a long history of the HercepTest^®^ being successfully used to select patients [179], and there is a similar history in the development of FRα diagnostics [180]. However, it may also reflect the relatively poor correlation between antigen expression levels and clinical efficacy for other approved ADCs such as Polivy^®^ [87].

### 3.2. Toxicities Associated with Target Expression on Non-Malignant Tissues

Whilst toxicities observed clinically with ADC treatments are most commonly due to off-target effects (e.g., through premature release of the payload), on-target toxicities can also present a significant challenge. In particular, the on-target off-tumour toxicity of an ADC can be influenced by the choice of target antigen.

In order to mitigate toxicity, the physiological role of a target antigen and the mechanisms by which it fulfils this role must also be considered. Therefore, pre-clinical toxicity studies for a novel ADC target not only require a study of the differential expression of the target between tumour and normal tissues but also require an investigation of the physiological functions of the target in order to identify potential toxicities. The importance of differential target expression on normal versus malignant cells is usually evident from the toxicity mediated by on-target off-tumour antigen expression observed during the clinical evaluation of ADCs. For example, a Phase I trial of the anti-CD44 antibody bivatuzumab conjugated to DM-1 for the treatment of squamous cell carcinoma reported a fatal skin toxicity, potentially attributable to the expression of CD44 by healthy keratinocytes [181].

A comparison of the distribution of the antigens targeted by the approved ADCs on normal and malignant cells with the commonly observed Grade 3 or above TEAEs for these ADCs (Table 4) provides insights into the importance of antigen expression distribution. While common TEAEs observed in the clinical trials of several ADCs are related to myelosuppression and hepatotoxicity, it is interesting to note that there are only two target antigens, HER2 and Nectin-4, for which potential causal links have been established between the expression of antigen on normal cells and in cardiomyocytes and skin, respectively, and the respective cardiotoxicity and skin rash observed in clinical evaluations [120,121,122,155].

However, although expression of a target antigen by normal cells is a significant factor for consideration, it does not necessarily impede the development and ultimate success of an ADC. For example, in safety studies on an ADC comprised of an anti-NaPi2b antibody against the solute carrier encoded by SLC34A2 conjugated to MMAE, an acceptable level of toxicity was observed in non-human primates despite high expression of the target antigen in normal primate lung [210]. In another example, despite high levels of expression of TROP-2 in some normal tissues, Trodelvy^®^ was successfully developed and approved by the FDA for the treatment of metastatic triple-negative breast cancer [137,138,211]. It has been hypothesised that for an anti-TROP-2 ADC, the lower expression of antigen in normal tissues compared to malignant tissues may be sufficient to avoid serious toxicities. Alternatively, it has been suggested that intracellular rather than cell surface expression of TROP-2 on normal cells, or expression at sites such as the luminal sides of ductal or glandular epithelia, which are not accessible by an antibody or an ADC, may play a role [212].

The viability of an ADC targeting a tumour antigen that is also highly expressed on normal cells may be enhanced through ADC design strategies. One such strategy is the development of probody-drug conjugates, an approach that has been applied to a checkpoint inhibitor therapy evaluated in early-stage clinical trials [213]. In this approach, the antigen-binding sites of the antibody are blocked by peptides and only exposed at tumour sites through the action of native tumour-associated proteases. For example, this mechanism has allowed the targeting of abundantly expressed CD71 (Transferrin Receptor 1) by the anti-CD71-MMAE probody-drug conjugate CX-2029, which has been evaluated in a Phase I clinical trial [214].

### 3.3. Significance of Antigen Shedding on ADC Function

When selecting an ADC target, the rate of shedding of the antigen may be an important consideration. In the context of antibody-based therapeutics, antigen shedding refers to the removal of target antigens expressed on the surface of cells, a process often mediated by proteases, as a means of functional regulation [50]. Early studies on immunotoxins suggested that increased antigen shedding could be detrimental for the effectiveness of ADCs by reducing the amount of ADC available for on-target, on-tumour binding [50]. However, other studies using mathematical and experimental models implied that high rates of antigen shedding may increase or decrease ADC effectiveness, contingent on several factors including rates of ADC endocytosis, ADC recycling, and extravasation through the tumour microenvironment.

**Figure 4:**
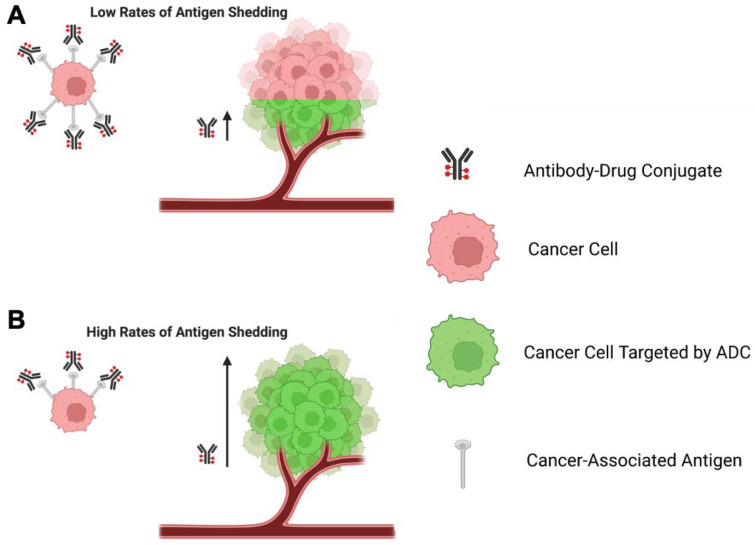
Schematic representation of the proposed effect of antigen shedding on the binding-site barrier and ADC effectiveness. (**A**) When rates of antigen shedding are low, a greater number of cell surface antigens per cancer cell are available which serve as a ‘binding-site barrier’, depleting the supply of ADC molecules entering the tumour microenvironment close to the vasculature. (**B**) When rates of antigen shedding are high, a smaller number of cell surface antigens per cancer cell are available, meaning that fewer ADCs bind to each cancer cell and a greater number of ADC molecules are available to bind to more cancer cells distant from the vasculature. Created with Biorender.com.

The development of a mathematical model that simulated tumour growth after administration of an anti-mesothelin immunotoxin suggested that the sink effect potentiated by a high rate of antigen shedding may be compensated for by a high degree of ADC recycling to the membrane post-internalisation. This recycling may generate a reservoir of functional ADC. Under such conditions, the model suggested that high rates of shedding may actually increase immunotoxin restriction of tumour growth, possibly by reducing the repeat binding of immunotoxin to previously targeted tumour cells [53]. This might allow the immunotoxin to move more rapidly through the tumour microenvironment without being slowed down by constant binding, a phenomenon known as the ‘binding-site barrier’ [53,215]. The proposed effects of high or low rates of antigen shedding on the binding site barrier are summarised in Figure 4.

The same model, refined and applied to the same anti-mesothelin immunotoxin as well as to an anti-CD25 immunotoxin, suggested that whether or not increased antigen shedding is advantageous can depend on the type of target antigens. In this case, an increased shedding rate was found to increase the effectiveness of the former immunotoxin but reduce the potency of the latter [52]. The differential effects of high shedding rates on immunotoxin efficacy have been postulated to be dependent on antigen-specific factors such as the number of binding sites available per cell and the rate of endocytosis of the immunotoxin [51]. Therefore, the mathematical models suggest that the propensity of high levels of antigen shedding to enhance or reduce ADC effectiveness can vary between antigens, a hypothesis consistent with experimental and mechanistic results.

An ADC target antigen for which the rate of shedding plays an important role is MUC16. As this antigen is overexpressed in ovarian cancer and other malignancies, ADCs targeting MUC16, such as sofituzumab vedotin, have been evaluated [216]. However, their limited success in the clinic to date may potentially be due to the well-characterised high levels of cleavage of the extracellular domain of MUC16, which may act as a sink for an ADC.

Antigen shedding may also play a direct role in signalling of the target antigen, as is the case for HER2. Shedding of the HER2 ectodomain results in the generation of a truncated subunit, p95HER2, associated with poorer clinical outcomes including lymph node metastasis in breast cancer, as well as with trastuzumab treatment resistance [113,217]. It has been established that an auxiliary property of Kadcyla^®^ is trastuzumab-mediated inhibition of the cleavage that generates p95HER2, thus conferring an anti-tumour effect by inhibiting pro-growth signalling via the truncated HER2 receptor [118]. This is supported by the observation that a high p95HER2:HER2 ratio has been associated with poorer outcomes for patients with HER2-positive metastatic breast cancer after treatment with trastuzumab [218]. Therefore, it is important to consider whether the shedding of an antigen may exert a pro-tumourigenic role that may be affected by ADC-antigen binding.

In summary, several studies have suggested that during the identification of an appropriate ADC target, antigen shedding should be considered, although it is not possible at present to predict its effect on a given antigen type.

## 4. Conclusions

An appraisal of the target antigens of approved and emerging ADCs against both solid tumours and haematological malignancies has revealed several factors that should be considered. Once a target antigen with sufficient differential expression on malignant versus normal cells has been identified, its suitability for developing an ADC against should be further evaluated by considering factors including (1) the physiological functions of the antigen in both normal and tumour cells, (2) whether, where and how the antigen is shed, and the potential impact of shedding on ADC effectiveness, (3) antigen recycling and its effect on ADC mechanism of action, (4) the extent of expression of the antigen in healthy organs and tissues, and (5) the kinetics and mechanism of trafficking of the physiological ligand upon binding to the cell surface antigen. These factors are summarised in Table 5. If antigen-mediated factors do not appear to preclude a given antigen from serving as an ADC target, then the trafficking pathways, expression pattern and physiological function of the antigen should next be considered to inform decisions relating to the isotype of the antibody chosen. Rigorous selection of ADC target antigens in this manner, as well as consideration of how the choice of antigen may affect other aspects of ADC performance, should help to produce optimised clinically-effective ADCs for the treatment of both solid and haematological malignancies.

## Figures and Tables

**Table 1:** Approved ADCs for the Treatment of Solid Tumours and Haematological Malignancies.

ADC Name	Developer	Year of Approval	Indication	Antibody Isotype	Target	Target Description	Payload	Linker Type	Approximate DAR	Payload Mechanism
**ADCs with Tubulin Inhibiting Payloads**										
Brentuximab vedotin (Adcetris^®^)	Seagen	2011 (FDA) 2012 (EMA)	HL, Systemic ALCL	IgG1	CD30	Marker of activated lymphocytes	MMAE	Cleavable	4	Tubulin inhibition
Ado-trastuzumab emtansine (Kadcyla^®^)	Genentech	2012 (FDA) 2013 (EMA)	HER2-positive breast cancer	IgG1	HER2	Growth Factor Receptor	DM-1	Non-cleavable	4	Tubulin inhibition
Polatuzumab vedotin-piiq (Polivy^®^)	Genentech	2019 (FDA) 2020 (EMA)	DLBCL	IgG1	CD79b	B-cell Receptor component	MMAE	Cleavable	3	Tubulin inhibition
Enfortumab vedotin-ejfv (Padcev^®^)	Astellas/Seagen	2019 (FDA*) 2022 (EMA)	Urothelial cancer	IgG1	Nectin-4	Adhesion Molecule	MMAE	Cleavable	4	Tubulin inhibition
Belantamab mafodotin-blmf (Blenrep^®^)	GlaxoSmithKline	2020 (EMA), 2020 (FDA*, To be withdrawn)	MM	IgG1	BCMA	Marker of mature B cells	MMAF	Non-cleavable	4	Tubulin inhibition
Tisotumab vedotin-tftv (Tivdak^®^)	Genmab/Seagen	2021 (FDA)	Cervical cancer	IgG1	Tissue factor	Blood Clotting Co-factor	MMAE	Cleavable	4	Tubulin inhibition
Mirvetuximab soravtansine-gynx (Elahere^®^)	ImmunoGen	2022 (FDA*)	Platinum-resistant, FRα-positive epithelial ovarian, fallopian tube, or primary peritoneal cancer	IgG1	FRα	Folic Acid Metabolic Receptor	DM-4	Cleavable	2	Tubulin inhibition
**ADCs with DNA-Interactive Payloads**										
Gemtuzumab ozogamicin (Mylotarg^®^)	Pfizer	2000 (Withdrawn) 2017 (FDA) 2018 (EMA)	AML	IgG4	CD33	Myeloid-specific marker	Calicheamicin	Cleavable	2	DNA Cleaving
Inotuzumab ozogamicin (Besponsa^®^)	Pfizer	2017 (EMA) 2017 (FDA)	ALL	IgG4	CD22	B-cell Receptor component and negative regulator of B-cell receptor signalling	Calicheamicin	Cleavable	6	DNA Cleaving
Trastuzumab deruxtecan-nxki (Enhertu^®^)	AstraZeneca/Daiichi Sankyo	2019, 2021, 2022 (FDA*) 2021 (EMA)	HER2-positive metastatic breast cancer, HER2-mutated NSCLC, HER2-positive gastric or gastroesophogeal cancer	IgG1	HER2	Growth Factor Receptor	Dxd	Cleavable	8	Topoisomerase inhibition
Sacituzumab govitecan-hziy (Trodelvy^®^)	Immunomedics/Gilead Sciences	2020 (FDA) 2021 (EMA)	TNBC, HR-Positive, HER2-negative breast cancer, Urothelial Carcinoma	IgG1	TROP-2	Transmembrane Glycoprotein	SN-38	Cleavable	8	Topoisomerase inhibition
Loncastuximab tesirine-lpyl (Zynlonta^®^)	ADC Therapeutics	2021 (FDA), 2022 (EMA)	DLBCL	IgG1	CD19	B-cell marker and positive regulator of B-cell receptor signalling	PBD Dimer	Cleavable	2	DNA Cross-Linking

ADCs marked with ‘*’ have been granted accelerated approval by the FDA. Blenrep^®^ is to be withdrawn from the US market. Abbreviations: ALCL, Anaplastic Large Cell Lymphoma; ALL, Acute Lymphoblastic Leukaemia; AML, Acute Myeloid Leukaemia; BCMA, B Cell Maturation Antigen; DAR, Drug Antibody Ratio; DLBCL, Diffuse Large B Cell Lymphoma; Dxd, Deruxtecan; DM-1, Maytansinoid DM-1; EMA, European Medicines Agency; FDA, US Food and Drug Administration; FRα, Folate Receptor Alpha; HER2, Human Epidermal Growth Factor Receptor 2; HL, Hodgkin Lymphoma; MM, Multiple Myeloma; MMAE, Monomethyl Auristatin E; MMAF, Monomethyl Auristatin F; NSCLC, Non-Small Cell Lung Cancer; TNBC, PBD, Pyrrolobenzodiazepine; Triple-Negative Breast Cancer; TROP-2, Trophoblast Antigen 2.

**Table 2:** Selected Novel ADCs in Late-Stage Clinical Development.

ADC Name	Developer	Stage	Clinical Trial Number	Indication	Antibody Isotype	Target	Target Description	Payload	Payload Mechanism
**ADCs with Tubulin Inhibiting Payloads**									
ARX788	Ambrx, NovoCodex Biopharmaceutical Co.	Phase II/III	CTR20200713	Metastatic Breast Carcinoma	IgG1	HER2	Growth Factor Receptor	Auristatin	Tubulin inhibition
Tusamitamab ravtansine	ImmunoGen, Sanofi, Innovent Biologics (Suzhou)	Phase III	NCT04154956	NSCLC	IgG1	CEACAM5	Cell Adhesion Molecule	DM-4	Tubulin inhibition
Trastuzumab emtansine (Ujvira^®^) *	Zydus Cadila *	Phase III	N/A	HER2-Positive Metastatic Breast Cancer	IgG1	HER2	Growth Factor Receptor	DM-1	Tubulin inhibition
Disitamab vedotin (Aidexi^®^)	RemeGen Co, Seagen	Phase IV	NCT05488353	Urothelial Carcinoma	IgG1	HER2	Growth Factor Receptor	MMAE	Tubulin inhibition
Upifitamab rilsodotin	Mersana Therapeutics	Phase III	NCT05329545	High-Grade Serous Ovarian Cancer	IgG1	NaPi2b	Sodium/Phosphate Transporter	Auristatin	Tubulin inhibition
Telisotuzumab vedotin	Abbvie	Phase III	NCT04928846	NSCLC	IgG1	c-Met	Growth Factor Receptor	MMAE	Tubulin inhibition
Zilovertamab vedotin	Velos Bio, Merck	Phase II/III	NCT05139017	Relapsed or refractory DLBCL	IgG1	ROR-1	Receptor Tyrosine Kinase-Like Receptor	MMAE	Tubulin inhibition
**ADCs with DNA-Interactive Payloads**									
SKB264	Klus Pharma, Merck	Phase III	NCT05347134	Locally Advanced, Recurrent or Metastatic TNBC	Undisclosed	TROP2	Transmembrane Glycoprotein	Belotecan Derivative	Topoisomerase inhibition
Datopotamab deruxtecan	Daiichi Sankyo, AstraZeneca	Phase III	NCT05104866	HR-positive, HER2-Negative Breast Cancer	IgG1	TROP-2	Transmembrane Glycoprotein	Dxd	Topoisomerase inhibition
Patritumab deruxtecan	Daiichi Sankyo	Phase III	NCT05338970	NSCLC	IgG1	HER2	Growth Factor Receptor	Dxd	Topoisomerase inhibition
Pivekimab sunirine (IMGN632)	ImmunoGen	Phase I/II	NCT03386513	BPDCN, AML	IgG1	CD123	IL-3 Receptor	DGN549	DNA Guanine Mono-Alkylation
Trastuzumab duocarmazine	Byondis	Phase III	NCT03262935	Metastatic Breast Cancer	IgG1	HER2	Growth Factor Receptor	Duocarmycin	DNA Adenine Mono-Alkylation
Trastuzumab rezetecan	Jiangsu HengRui Medicine, Luzsana Biotechnology	Phase III	NCT05424835	HER2-Positive Metastatic Breast Cancer	IgG1	HER2	Growth Factor Receptor	Camptothecin	Topoisomerase inhibition
Vobramitamab duocarmazine	Byondis, MacroGenics	Phase II/III	NCT05551117	Prostate Cancer	IgG1	B7-H3	Immunoregulatory Glycoprotein	Duocarmycin	DNA Adenine Mono-Alkylation

* Ujvira^®^ is the first biosimilar of trastuzumab emtansine (Kadcyla^®^) to be approved by the Drug Controller General of India (DCGI). Abbreviations: AML, Acute Myeloid Leukaemia; BPDCN, Blastic Plasmacytoid Dendritic Cell Neoplasm; CEACAM5, Carcinoembryonic Antigen Cell Adhesion Molecule 5; DM-1, Maytansinoid DM-1; DM-4, Maytansinoid DM-4; HER2, Human Epidermal Growth Factor Receptor 2; HR, Hormone Receptor; NSCLC, Non-Small Cell Lung Cancer; TROP-2, Trophoblast Antigen 2.

**Table 3:** **Comparative** features of Enhertu^®^ and Kadcyla^®^.

Feature	Ado-Trastuzumab Emtansine (Kadcyla^®^)	Trastuzumab Deruxtecan (Enhertu^®^)
**Antibody**	Trastuzumab	Trastuzumab
**Target Antigen**	HER2	HER2
**Linker Type**	Non-cleavable	Cleavable
**Payload**	DM-1	Dxd
**Payload Mechanism**	Tubulin inhibition	Topoisomerase inhibition
**Drug Antibody Ratio (DAR)**	3.5	7.7
**Approved Indications**	HER2-positive breast cancer	HER2-positive metastatic breast cancer, HER2-mutated NSCLC, HER2-positive gastric or gastroesophageal cancer

Abbreviations: DM-1, Maytansinoid DM-1; Dxd, Deruxtecan; HER2, Human Epidermal Growth Factor Receptor 2; NSCLC, Non-small-cell lung carcinoma.

**Table 4:** Tissue Distribution of Target Antigens and Associated Toxicities. with ADCs.

Antigen	Cancer Types with High Expression	Normal Tissue Distribution	Commonly Observed Toxicities for ADC Treatment
CD33	AML [60]	Salivary Gland Kidney Epididymis Spleen Lymph Node Tonsil Testis Duodenum Bone Marrow	**Gemtuzumab ozogamicin (Mylotarg^®^)** Hepatic Veno-occlusive Disease [62] Elevated Aspartate/ Alanine Aminotransferase [182] Hyperbilirubinemia [182] Pneumonia [182] Dyspnoea [182] Neutropenia [182,183] Thrombocytopenia [183] Fever [182,183] Chills [182,183] Nausea [182] Hypertension [182] Hypotension [182] Sepsis [182]
**CD22**	B-ALL [184] B-NHL [185]	Appendix Lymph Node Testis Spleen Tonsil	**Inotuzumab ozogamicin (Besponsa^®^)** Hepatic Veno-occlusive disease [75] Thrombocytopenia [75] Febrile Neutropenia [75] Pneumonia [75]
**CD19**	B-NHL [185]	Bone Marrow Appendix Spleen Lymph Node Tonsil	**Loncastuximab tesirine (Zynlonta^®^)** Neutropenia [82,186,187] Anaemia [82,186,187] Thrombocytopenia [82,186,187] Lymphopenia [82,186] Leukopenia [82] Elevated gamma-glutamyl transferase [82,186,187] Elevated Blood Alkaline Phosphatase [82,186,187] Hypokalaemia [82,186] Hypophosphatemia [82] Fatigue [187] Dyspnoea [82]
**CD79b**	B-NHL [83]	Appendix Spleen Lymph Node Tonsil	**Polatuzumab vedotin (Polivy^®^)** Anaemia [89,188] Neutropenia [89,188] Lymphopenia [89] Thrombocytopenia [89]
BCMA *	MM [189] CLL [189] B-ALL [189] B-NHL [189] HL [189]	Plasma Cells [94] Mature B Cells [94]	**Belantamab mafodotin (Blenrep^®^)** Keratopathy [34] Thrombocytopenia [34] Anaemia [34] Nausea [34] Pyrexia [34] Blurred Vision [34] Increased Aspartate Aminotransferase [34] Fatigue [34] Dry Eyes [34] Neutropenia [34] Hyperkalaemia [34] Lymphopenia [34] Increased Gamma Gliutamyltransferase [34] Hypophosphataemia [34] Pneumonia [34]
**CD30**	HL [100] CTCL [190] PTCL-NOS [190]	Testis Fallopian Tube Appendix Lymph Node Tonsil	**Brentuximab vedotin (Adcetris^®^)** Neutropenia [191] Peripheral Neuropathy [191]
**HER2**	Biliary Tract Cancer [107] Colorectal Cancer [107] NSCLC [107] Breast Cancer [108] Gastric Cancer [192]	Nasopharynx Lung Urinary bladder Testis Fallopian Tube Endometrium Cervix Placenta Breast Heart muscle Skeletal muscle Skin Appendix Thyroid gland Parathyroid Gland Bronchus Salivary gland Oesophagus Small intestine Rectum Liver Kidney Seminal vesicle Prostate Vagina Tonsil Bone marrow Cerebral cortex	**Trastuzumab emtansine (Kadcyla^®^)** Thrombocytopenia [125,126,127,193] Elevated Aspartate Aminotransferase [127] **Trastuzumab deruxtecan (Enhertu^®^)** Nausea [128,130,194] Fatigue [128,130,194] Neutropenia [128,130,194] Anorexia [128] Anaemia [128,130,194] Lymphopenia [128] Leukopenia [128] Thrombocytopenia [130]
**TROP-2**	Breast, Pancreatic, Urothelial and Ovarian Squamous Cell Carcinomas [138]	Nasopharynx Bronchus Oral mucosa Oesophagus Kidney Urinary bladder Seminal vesicle Cervix Skin Salivary gland Rectum Gallbladder Pancreas Epididymis Vagina Fallopian tube Endometrium Breast Tonsil	**Sacituzumab govitecan (Trodelvy^®^)** Nausea [145,195] Neutropenia [147,152,195] Leukopenia [147,152] Lymphopenia [152] Anaemia [152,195] Fatigue [145,195] Diarrhoea [145,152,195] Hypophosphatemia [145]
**Nectin-4**	Bladder Cancer [155] Breast Cancer [155] Pancreatic Cancer [155] Ovarian Cancer [155] Head and Neck Cancer [155] Oesophageal Cancer [155]	Oral mucosa Oesophagus Urinary bladder Placenta Breast Skin Tonsil Pancreas Kidney Testis Epididymis Seminal vesicle Prostate Vagina Endometrium Cervix	**Enfortumab vedotin (Padcev^®^)** Fatigue [196] Maculopapular Rash [196,197] Neutropenia [196,197] Anaemia [197] Diarrhoea [197] Hyperglycaemia [197,198] Increased Lipase [197] Anorexia [197]
TF *	Glioblastoma [166,199] Breast Cancer [161,199,200] Colorectal Cancer [166,199] Pancreatic Cancer [166,199] Lung Cancer [161] Cervical Cancer [201,202]	Monocytes [203] Platelets [203] Endothelial Cells [204]	**Tisotumab vedotin (Tivdak^®^)** Fatigue [205] Nausea [205] Vomiting [205] Abdominal Pain [205] Anaemia [205] Hypokalaemia [205]
FRα *	Ovarian Cancer [166,168] Lung Cancer [166] Breast Cancer [166]	Fallopian Tube Epithelium [206] Placental Epithelium [207] Kidney [208] Lung [208]	**Mirvetuximab soravtansine (Elahere^®^)** Diarrhoea [170,171] Blurred Vision [170,171] Keratopathy [170,171]

Expression data for normal tissues that express each target antigen were compiled using the Human Protein Atlas (www.proteinatlas.org) [209]. Where data from the Human Protein Atlas were unavailable, target antigens are marked with *, and tissues and cell types in which the expression of each antigen is reported at the protein level are listed. Commonly observed toxicities are toxicities that were observed in clinical trials of a given ADC at Grade 3 or above, and in 5% or more patients. Abbreviations: ADC, Antibody-Drug Conjugate; B-ALL, B Cell Acute Lymphoblastic Leukaemia; B-NHL, B Cell Non-Hodgkin Lymphoma; BCMA, B Cell Maturation Antigen; CLL, Chronic Lymphoblastic Leukaemia; CTCL, Cutaneous T Cell Lymphoma; FRα, Folate Receptor Alpha; HER2, Human Epidermal Growth Factor Receptor 2; HL, Hodgkin Lymphoma; IHC, Immunohistochemistry; NSCLC, Non-Small Cell Lung Cancer; PTCL-NOS, Peripheral T Cell Lymphoma Not Otherwise Specified; TF, Tissue Factor; Trop2, Trophoblast Antigen 2.

**Table 5:** Summary of Antigen-Related Features that may affect ADC Effectiveness.

Antigen-Related Feature	Factors Increasing ADC Effectiveness	Factors Reducing ADC Effectiveness	Examples of Relevant Antigens
Physiological Functions of the Target Antigen	If the target antigen promotes pro-tumour signalling pathways, targeting with an ADC may also exert antitumour effects by inhibiting oncogenic signalling pathways.	If signalling mediated by the target antigen is required for essential normal functions, inhibition of this signalling and killing of non-malignant cells expressing the antigen may lead to toxicity.	**Antigens Promoting Pro-Tumour Signalling Pathways** BCMA [94] CD30 [100,102] HER2 [108] TROP-2 [139,140] FRα [166] **Antigens that when targeted by an ADC lead to target-mediated toxicity by inhibiting essential normal functions** HER2 [120,121,122]
Antigen Shedding	Reduction in ‘Binding-Site Barrier’ for greater penetration of ADC.	‘Sink effect’ potentiated by ADC binding to shed antigen.	**Highly Shed:** HER2 [113,118,217] Nectin-4 [156] CD25 [219] MUC16 [220] TROP-2 [140] FRα [168] **Limited Shedding:** CD19 [41] CD22 [73]
Recycling of Antigen to the Plasma Membrane	A high degree of ADC recycling could counteract the sink effect caused by high rates of antigen shedding.	Direction of internalised ADC to the plasma membrane without potentiation of cytotoxicity.	**Antigens which may be recycled to the plasma membrane:** CD22 [73] HER2 [116] TROP-2 [142]
High Expression on Malignant Cells, Low or Absent Expression on Normal Cells	Therapeutic window for targeting malignant cells while sparing healthy cells.	-	**Antigens for which this paradigm does not appear to directly apply:** TROP-2 [137,139] SLC34A2 [210] HER2 [174] CD70 [176]
Rapid *de novo* synthesis of antigen and restoration of cell surface expression	More rapid repeat targeting of the same cell by an ADC	*-*	**Antigens rapidly synthesised *de novo:*** CD33 [58]
